# The Great American Medical Regulator

**Published:** 1857-10

**Authors:** 


### The Great American Medical Regulator.

We believe it is generally understood that Dr. Bowling of the Nashville Journal is the *self constituted regulator* of the medical affairs of the United States of America.

We believe that it is equally well known that his Journal is, and has been, the continual vehicle for all manner of unjust and foul aspersions, directed against those who dared oppose his presumptuous claims. Under these circumstances, we hardly think it necessary to reply to the unprovoked *personal* discourtesy contained in his last number.

